# Development of a procedure for isolation, identification and quality assessment of bovine spermatids and evaluation of their fertilizing ability *in vitro*

**DOI:** 10.3389/fbioe.2025.1581019

**Published:** 2025-05-16

**Authors:** Rolando Pasquariello, Francesca Di Filippo, Sai Kamal Nag Bonumallu, Fernanda Fagali Franchi, Ramona Pistucci, Federica Franciosi, Valentina Lodde, Alessandra Iannuzzi, Alberto Maria Luciano, Tiziana A. L. Brevini, Fulvio Gandolfi

**Affiliations:** ^1^ Department of Agricultural and Environmental Sciences - Production, Landscape, Agroenergy, Università degli Studi di Milano, Milan, Italy; ^2^ Reproductive and Developmental Biology Laboratory, Department of Veterinary Medicine and Animal Sciences, Università degli Studi di Milano, Lodi, Italy; ^3^ Institute for Animal Production System in Mediterranean Environment, National Research Council, Naples, Italy; ^4^ Laboratory of Biomedical Embryology and Tissue Engineering, Department of Veterinary Medicine and Animal Sciences, Università degli Studi di Milano, Lodi, Italy

**Keywords:** cattle, bull testis, spermatids, intracytoplasmic spermatid injection, flow cytometry cell sorting, embryotechnology

## Abstract

Intracytoplasmic spermatid injection into oocytes has limited efficiency in cattle, with no offspring generated so far, partly due to ambiguous spermatid identification. This study aimed to develop and validate a method for isolating and characterizing bovine spermatids to improve the efficiency of spermatid intracytoplasmic injection. First, we optimized a protocol for spermatid isolation from bull testis using a discontinuous Percoll gradient and 10 μm mesh cell strainers. Next, we established a stage-specific separation strategy based on DNA content, size, and granularity using flow cytometry to distinguish round and elongating/elongated spermatids suitable for molecular analysis. Morphological assessment confirmed that 72.5% of isolated cells were at the spermatid stage, supported by a high haploidy rate, spermatid-specific transcript expression (*PRM1*, *PRM2*, *SPACA9*, *SPERT*), and SPERT protein detection. Viability assays showed that spermatids maintained intact DNA at 0 and 24 h at 4°C and 37°C, though mitochondrial activity and ROS levels increased over time, suggesting oxidative stress. When spermatids were injected into oocytes (n = 82), only 13.4% formed two pronuclei, whereas 46.3% exhibited a single pronucleus and a condensed chromatin spot, indicating incomplete activation or fertilization failure. This work contributes to refining bovine intracytoplasmic injection protocols. Future applications of this approach, particularly if functional spermatids can be derived from spermatogonia or embryonic cells, could help shorten the generational interval in cattle breeding.

## 1 Introduction

Modern agricultural advancements have transformed livestock production, increasing the need for efficient breeding strategies (Ritchie and Roser, 2024). In cattle, optimizing genetic selection is crucial to meet the rising global demand for food (Henchion et al., 2021), while improving disease resistance, climate resilience, and animal welfare (Berglund, 2015). One possible approach to enhance breeding efficiency is by shortening the generation interval, which can accelerate genetic improvement and adaptability (Pasquariello et al., 2024).

Genomic selection (GS) has been widely adopted in livestock breeding programs to accelerate genetic gain, boosting production efficiency and promoting the sustainability of animal agriculture. When GS is combined with Assisted Reproductive Techniques (ARTs), also known as embryotechnology, the highest increase in genetic gain is achieved by reducing the generation interval (Cenariu et al., 2012). Multiple Ovulation Embryo Transfer (MOET), also known as *in vitro* derived embryos (IVD) (Reuben and Bó, 2020), allows increased female selective pressure by reducing the generation interval to approximately 1 year (Lafontaine et al., 2023). The emerging frontiers of generating functional male and female gametes *in vitro* from neonatal gonads or even embryonic cells are paving the way for an innovative approach that could shorten the generational interval to a few weeks (Hayashi et al., 2012; Goszczynski et al., 2019). This strategy involves using immature cells, such as round and elongated spermatids, for intracytoplasmic injection into *in vitro* matured (IVM) oocytes. Spermatid injection has been successfully used in human (Tanaka et al., 2015) and mouse (Zhu et al., 2024), leading to full-term offspring. However, its use in cattle is still limited due to difficulties in identifying, retrieving and handling spermatids, and low fertilization and embryo development rates (Ock et al., 2006a; 2006b).

This work aimed to develop a protocol for isolating bovine spermatids from bull testis and to characterize their morphological features for accurate identification. Additionally, we aimed to confirm spermatid haploidy, assess the expression of key spermatid markers, and evaluate their viability and quality under different culture conditions. Furthermore, we sought to assess the fertilizing ability of isolated spermatids and establish a flow cytometry procedure for stage-specific spermatid isolation based on DNA content, size, and granularity.

## 2 Materials and methods

All reagents were purchased from Sigma-Aldrich, Milan, Italy, unless otherwise indicated. If not stated, each experiment was performed in 5 replicates.

### 2.1 Ethical statement

Bovine testes were collected from an authorized local slaughterhouse. This study did not involve the use of living animals; therefore, ethical approval was not required.

### 2.2 Spermatid isolation protocol

**FIGURE 1:**
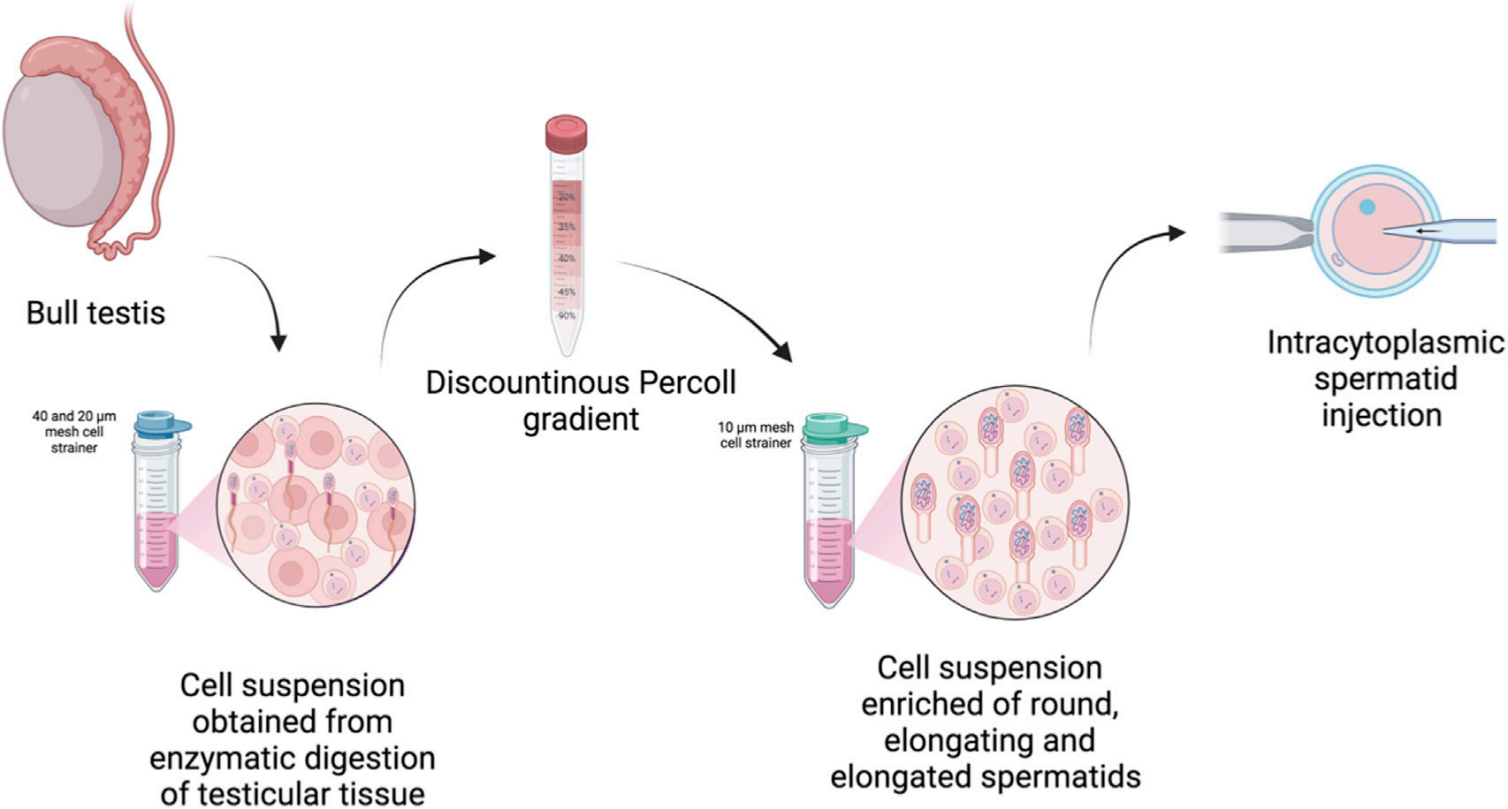
Overview of the procedure used to isolate bovine spermatids. Created in BioRender: https://BioRender.com/3rs70m1.

Testes were collected from 10 mature Charolaise bulls (24 months of age), destined for meat production, immediately after slaughtering. Testis were transported on ice to the laboratory within 1 h (h) to ensure optimal tissue preservation. After testis decapsulation, testicular parenchyma was washed twice with Dulbecco’s phosphate-buffered saline (DPBS). Thereafter, 10 mg of tissue was minced into small pieces with scalpels. Tissue pieces were then incubated into 20 mL Dulbecco’s Modified Eagle Medium/Nutrient Mixture F-12 (DMEM/F12, Thermofisher) containing 0.2 mg/mL Pronase at 37°C for 3 min. To stop enzyme activity, 10 mL DMEM supplemented with 10% fetal bovine serum (FBS, Gibco) were added to the digested tissue pieces. Thereafter, cell clumps and debris and obtained cell suspension were filtered using 40 and 20 µm nylon mesh cell strainers. Then, the tube was centrifuged at 500 × g for 5 min (min) to precipitate the cells. The cell pellet was washed twice with DPBS followed each time by centrifugation at 500 × g for 5 min. The cell pellet was then resuspended using 0.5 mL DMEM/F12 and layered on a discontinuous Percoll gradient made of 2 mL each of 90%, 45%, 40%, 35% and 20% Percoll solutions diluted with DMEM/F12 using a 15 mL conical tube as described in previous work (Ock et al., 2006b; 2006a). The tube was centrifuged for 25 min at 650 x g. After centrifugation, 35% and 40% Percoll gradients were collected and washed three times using 5 mL DPBS and once using 1 mL DMEM/F12 supplemented with 10% FBS. Cell suspension was then filtered using a 10 µm nylon mesh cell strainer. After isolation, cells were immediately analysed or used for round spermatid injection. In some experiments, their quality was assessed after 24 h of culture. An overview of the isolation procedure is shown in Figure 1.

### 2.3 Spermatid identification and characterization

#### 2.3.1 Morphological evaluation and identification

Spermatids were identified by morphological assessment upon staining with Haematoxylin and Eosin or 2-(4-amidinophenyl)-1H -indole-6-carboxamidine (DAPI) (Thermofisher). To this end, cells were washed 2–3 times using 2 mM Ethylenediaminetetraacetic acid (EDTA) in phosphate-buffered saline (PBS) and diluted in 0.2 mL PBS 1% BSA containing 30,000–50,000 cells and spin onto slides for 5 min at 112 x g. The obtained slides were air dried for 10 min, fixed using PFA 4% and stored ad 4°C in PBS.

#### 2.3.2 Assessment of haploidy using FISH analysis

Isolated spermatids were washed twice in 6 mM EDTA PBS and fixed using 3:1 methanol: acetic acid solution. A 5 µL droplet of fixed cell suspension was dropped on a clean microscope slide and air dried at room temperature. DNA was denaturation was performed by submerging each slide in 3 M sodium hydroxide at room temperature for 2 min, facilitating DNA decondensation. Thereafter, the slides were washed in distilled water, dehydrated by sequential submersion in 70, 80, and 96% ethanol solutions (for 2 min each), and air-dried before hybridization following the protocols described by Iannuzzi et al. (2023). The probes used in the study consisted of pools of three Bacterial Artificial Chromosomes (BACs), selected as a contig to cover approximately 1 megabases of sequence, enhancing FISH signal intensity (Table 1). Probe preparation and dual-color FISH analysis were carried out according to the protocols described of Genualdo et al. (2021), with slight modifications. Briefly, probes were labeled using the Biotin and Dig-Nick Translation Mix kit (Roche Applied Science) as detailed in Table 1. They were denatured at 70°C for 10 min, then pre-annealed at 37.0°C for 60.0 min. For each slide, two probes were simultaneously hybridized to the decondensed sperm heads, covered with 24-mm coverslips, sealed, and incubated at 37°C in the humidified chamber for 24 h. Post-hybridization washes included two 5-minute incubations in 50% formamide in 2X saline-sodium citrate (SSC) at 45°C, followed by two additional 5-minute washes in 2X SSC at 45°C, following the protocols described by Dio et al. (2020). Hybridization sites were detected using indirect labeling FITC-avidin for the biotinylated probes and an anti-digoxigenin antibody conjugated to a red fluorophore for the digoxigenin-labeled probes. Slides were incubated for 1 h in the dark, humid chamber at 37°C for 1 h. After staining, they were counterstained with Vectashield DAPI H-1000 antifade solution (Vector Laboratories).

**TABLE 1:** Bacterial artificial chromosome (BAC) probes with their genome view (according to NCBI) used for dual colour FISH analysis. For each probe, total length in base pairs (bp), genome localization, label and imposed color are reported.

Probe	Total length (bp)	Bos taurus UMD 3.1.1 Genome localization (bp)	Label	Imposed color
1st pool				
bI 0004H06	83.78	22,452,603–22,536,386	Digoxigenin	Red
bI 0696G12	122.39	23,090,214–23,212,604	Digoxigenin	Red
bI 0042A07	242.34	23,287,629–23,529,972	Digoxigenin	Red
2st pool				
bI 0039B11	100.79	44,410,860–44,511,655	Biotin	Green
bI 0393A06	147.11	43,921,537–44,068,649	Biotin	Green
bI 0651C08	102,98	44,560,941–44,663,929	Biotin	Green

**FIGURE 2:**
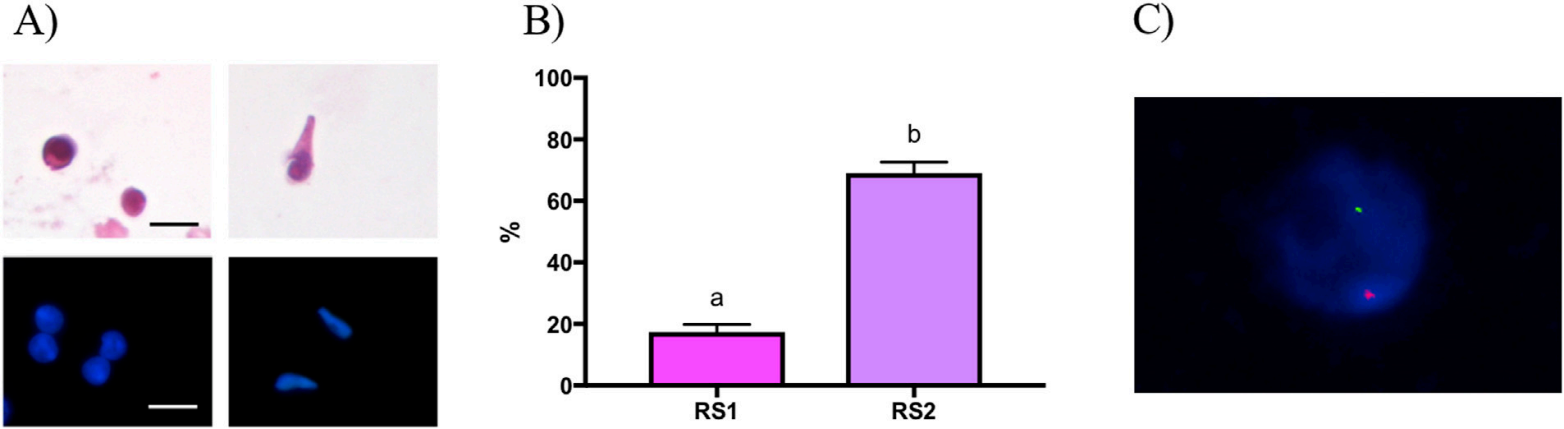
Identification of spermatids by morphological and haploidy evaluation. **(A)** Representative picture of isolated round and elongated spermatids stained with Hematoxylin and Eosin (top) and DAPI (bottom). **(B)** Results of morphological analysis performed to assess the percentage of spermatid after enzymatic digestion of testicular tissue (RS1) and after their isolation using the procedure described in Figure 1 (RS2). **(C)** Representative picture of nucleus stained using DAPI (blue) and FISH chromosome signals (red and green dots) for identification of haploid spermatids.

Slides were analyzed using a Leica DM 5500B fluorescence microscope equipped with a 100X objective, specific triple-bandpass filters (DAPI, FITC, Orange), and a high-sensitivity monochrome camera. Digital images were acquired in grayscale, and false-color images were generated using Cytovision-Leica software to differentiate signal types. Haploid spermatids were identified when a single signal was observed for each probe (Figure 2C).

#### 2.3.3 Gene expression analysis

Gene expression analysis was carried out by reverse transcription quantitative real-time PCR (RT-qPCR) for the following genes (Table 2): protamine 1 (*PRM1*), protamine 2 (*PRM2*), Spermatid-associated protein (*SPERT*), sperm acrosome associated 9 (*SPACA9*). A total of three biological replicates for each group (RS1 and RS2) were used for RT-qPCR. RNA extraction was carried out using TRIZol (15596018, Thermofisher) reagent combined to PureLink RNA Mini Kit (12183018A, Thermofisher) with on column DNAse treatment using the PureLink DNase (12185010, Thermofisher). Complementary DNA (cDNA) was synthesized using iScript Advanced cDNA Synthesis Kit for RT-qPCR (Bio-Rad, USA) following the manufacturer’s protocol**.** The cDNA samples were diluted 1:3 using RNase-free water and stored at −20°C until RT-qPCR was run. Each PCR reaction was performed in triplicate using 10 μL SsoAdvanced™ Universal SYBR Green Supermix (1725271, BioRad), 1.2 μL 10 μM primer mix, 6.8 μL nuclease free water and 2 μL 1:3 diluted cDNA sample using 96 well plates (HSP9601, Bio-Rad). The RT-qPCR program was run on a Bio-Rad CFX96™ Real time machine (Bio-Rad), as follows: 95°C for 30 s cycle followed by 40 cycles of amplification step at 95°C for 15 s and 59°C for 30 s. A melting curve was analysed for each experiment to assess the specificity of primer amplification. Relative gene expression was calculated using the 2^−ddCt^ method (Pfaffl, 2001). Normalization of Ct values was obtained using the expression of glyceraldehyde-3-phosphate dehydrogenase (*GAPDH*) and beta actin (*ACTB*).

**TABLE 2:** List of primers used for real time quantitative PCR analysis. For each reverse and forward primer, the name ID of the gene. The accession number and the forward and reverse sequences are reported.

Name ID	Accession number ID	Forward primer (5’->3′)	Reverse primer (5’->3′)
*PRM1*	NM_174,156.2	CAG​CCC​ACA​AAT​TCC​ACC​T	TGAGGCGCATCGGTATCT
*PRM2*	NM_174,157.4	CGC​TAC​CAC​TAC​AGA​CAC​AG	AAG​CTT​AGA​GCT​GCC​TTC​C
*SPERT*	NM_152,719.3	CTG​AAA​TGT​AGG​GTG​GAG​GAA​TC	GTA​CAA​GTT​GTG​GAG​CCT​CAG
*SPACA9*	NM_001101193.2	GAACGCCCACGACAAGAT	GCA​GAT​GTC​CAG​GAA​CAT​GA
*GAPDH*	NM_001034034.2	TCA​TCA​TCT​CTG​CAC​CTT​CTG	ATG​CCA​AAG​TGG​TCA​TGG​A
*ACTB*	NM_173,979.3	TCT​TCC​AGC​CTT​CCT​TCC​T	TAG​AGG​TCC​TTG​CGG​ATG​T

#### 2.3.4 Immunofluorescence of spermatid associated protein (SPERT)

After spinning cells onto the slides using cytospin following the procedure described above, they were immediately fixed in 4% paraformaldehyde for 30 min at room temperature. Cells were then permeabilized with 0.1% Triton X-100 in PBS for 30 min at room temperature. Aspecific bindings were prevented by incubating cells in 10% goat serum in PBS for 30 min at room temperature. Afterwards, cells were stained using anti-SPERT primary antibody (1:150 dilution, ab243570, abcam) overnight at 4°C. Subsequently, cells were incubated with secondary antibody Alexa Fluor™ 594 goat anti-rabbit (1:250, A11012, Life Technologies Corporation) for 30 min at room temperature. Nuclei were counterstained with DAPI for 15 min at room temperature. Images were acquired using an Eclipse TE200 microscope (Nikon).

### 2.4 Assessment of spermatid quality during *in vitro* culture

#### 2.4.1 *In vitro* culture of spermatids

Quality assessment was carried out on the cells collected immediately after isolation and after 24 h of culture. In details, spermatids were either stored at 4°C in air atmosphere or cultured in DMEM/F12 medium supplemented with 10% FBS at 37°C in 5% CO_2_. Cell quality was examined scoring cell viability (LIVE/DEAD staining), DNA integrity (acridine orange), and mitochondrial activity (red CMXRos and green FM mitoTrackers) and analysis of reactive oxygen species (Image-iT Live Green ROS).

#### 2.4.2 DNA integrity

Isolated cells were smeared on polylysine slides, air dried and fixed in alcoholic solution (methanol CH_3_OH and acetic acid CH3COOH 3:1) overnight. Acridine orange (10 mg/mL) solution pH 2.5 was prepared with 0.1 M of Citric Acid and 0.3 M of Na_2_HPO_4_. Slides were stained with acridine orange solution for 10 min and air-dried. Then cells were observed under a fluorescence microscope.

#### 2.4.3 Cell viability

LIVE/DEAD™ Cell Imaging Kit (488/570) (R37601, Invitrogen) was used to determine the number of viable cells based on a sensitive two-color fluorescence cell viability assay optimized for evaluating intracellular esterase activity and plasma membrane integrity. Viability was quantified by dividing the number of live cells by the total number of cells and showed as a percentage of live and dead cells.

#### 2.4.4 Determination of reactive oxygen species

ROS concentration in single spermatid was measured using the Image-iT Live Green ROS detection kit (Invitrogen). Briefly, cells were washed three times in PBS. Cells were then stained with 25 μM 5- and 6-carboxy-20,70- dichlorodihydrofluorescein diacetate (carboxy-H2-DCFDA) in the dark for 30 min. Subsequently, cells were washed three times and immediately imaged using an Eclipse TE200 microscope (Nikon) with excitation of cells at 480 nm and emission at 510 nm. Image analysis was carried out using Fiji ImageJ (Pasquariello et al., 2018), and ROS concentration was calculated and reported as relative fluorescence units.

### 2.5 Intracytoplasmic injection into mature oocytes

#### 2.5.1 Oocyte collection and *in vitro* maturation (IVM)

Cumulus oocyte complexes (COCs) were isolated from ovaries, obtained from culled cows at a local slaughterhouse, by vacuum pump aspiration of 2–6 mm ovarian follicles, as previously described (Modina et al., 2007). Only COCs with at least 5 complete, compact cumulus layers and finely granulated ooplasm were selected for IVM culture in NaHCO_3_-buffered TCM199 supplemented with 0.68 mM L-glutamine, 0.4% fatty acid-free bovine serum albumin, 0.2 mM sodium pyruvate and 0.1 international units/mL of recombinant human follicle-stimulating hormone (r-hFSH, Gonal-F, Merck Serono SpA) in humidified air under 5% CO_2_ at 38.5°C for 22 h (Luciano et al., 2013). After 22 h, the cumulus cells were mechanically removed by gentle pipetting and cultured in the same IVM conditions for additional 2–4 h to allow recovery from the cumulus removal procedure and clearly visualize the extrusion of the first polar body (PBI).

#### 2.5.2 Spermatid injection into mature oocytes

The isolated spermatids were centrifuged to remove excess supernatant. A 2 µL aliquot of the spermatid suspension was added to one of the 5–6 droplets of 20 µL Hepes-buffered Tyrode’s albumin lactate pyruvate (TALP) on the microinjection dish. A 10 µL drop of 10% polyvinylpyrrolidone (PVP) (FertiCult) was placed at the center of the dish for priming the injection pipette, facilitating smooth injection. The dish was overlaid with OVOIL (Vitrolife), and the oocytes were transferred into the surrounding TALP droplets for injection. Spermatid injection was performed using a Nikon Ti2 Eclipse microscope equipped with a Narishige Takanome micromanipulator. Injection needles (Origio, Cooper Surgicals) with inner diameters (ID) 6.6–7.9 µm were used, along with holding needles (Kitazato) with an outer diameter (OD) of 120 µm. After loading the spermatids in the needle and orienting the oocyte with the polar body at 6 or 12 o’clock, the needle was inserted into the oocyte. The ooplasm was partially aspirated and released 2–3 times to ensure the oolemma was broken before depositing the spermatid into the cytoplasm. Oocytes were injected in batches of 10–15, and some were kept not injected as a control group.

#### 2.5.3 Oocyte activation and culture

All the injected oocytes and the not injected control group underwent a dual activation protocol (Horiuchi et al., 2002; Abdalla et al., 2009). First, they were incubated with 5 µM calcium ionophore (A23187, Sigma-Aldrich) for 5 min and cultured for 4 h in IVF medium (NaHCO_3_-buffered TALP) at 38.5°C, 5% CO_2_ in humidified air. Then a second activation with 7% ethanol for 5 min was applied, followed by 19–24 h culture in synthetic oviductal fluid (SOF) supplemented with 5% FBS at 38.5°C, 5% CO_2_, 5% O_2_ in humidified air. Finally, prospective zygotes were fixed in 4% paraformaldehyde, stained with DAPI-containing mounting media (Vectashield), and analyzed for two-pronuclei formation.

### 2.6 Fluorescent-activated sorting of spermatid populations by flow cytometry

In a different set of experiment, we aimed to separate the haploid spermatid subpopulations based on apparent DNA content, size, and granularity. In particular, we adapted the protocol originally described in the mouse (Simard et al., 2015). Briefly, testicular cells used for fluorescent-activated cell sorting (FACS) were obtained by digesting the testicular parenchyma using the digestion medium made with DMEM/F12 and pronase as described in 2.2. The obtained cell suspension was passed through a 40 µm nylon mesh cell strainer and washed 2–3 times using PBS before being resuspended into 3 mL PBS containing 2 mM EDTA pH 7. To fix isolated cells, 9 mL (3 volumes) of ice-cold 100% ethanol were slowly added using a 10 mL serological pipette with gentle agitation using a vortex at low speed. Then they were incubated for 15 min on ice and mixed every 3 min by inversion. Fixed cells were washed 3 times using a sorting buffer prepared with PBS containing 2 mM EDTA and 5% Serum Bovine Albumin. Cells were precipitated each time by centrifugation at 500 x g for 8 min. FACS was performed using BD FacsMelody - Cell Sorter. Briefly, cells were resuspended in 1 mL sorting buffer and stained using 15 µM SYTO 16 (cat.no. S7578, Invitrogen) for 30 min in the dark at 4 °C. An aliquot of unstained cells was kept separate to be used as a negative control. During cell sorting, the nozzle was set up to 100 μm, sort mode to 4 Way Purity with flow rate as 1.0 by which about 1,000 events per seconds were processed. Moreover, sample agitation was set up at 300 rpm and a temperature of 4 °C. Gating strategies were optimized to use a 488 nm laser-equipped cell sorter following the gating scheme detailed in Figure 6A and excluding small debris and cell doublets. Immediately after sorting, to carry out an epifluorescence microscopy evaluation of collected populations, collected cells were plated into 12-well plates, and nuclei were imaged using an Eclipse TE200 microscope (Nikon) with excitation at 480 nm and emission at 510 nm.

### 2.7 Statistical analyses

Before performing statistical analysis, data were checked for normality and homoscedasticity and it was confirmed that all the values were normally distributed. Subsequently, Fishers’ chi-square exact test and one-way ANOVA (SPSS 19.1; 240 IBM), followed by *post hoc* Tukey’s test, were performed depending on the number of experimental groups. All the results are expressed as the Mean ± Standard Error of the Mean (SEM), and differences in p-value ≤0.05 were considered statistically significant.

## 3 Results

### 3.1 Isolation of alive spermatids

#### 3.1.1 Identification of spermatids by morphological evaluation and fluorescent *in situ* hybridization

Morphological analysis revealed that round and elongated spermatids ranged between 7.0–12.0 μm in size. In round spermatids, nuclei were centrally located in the cell and had 1-3 nucleoli (Figure 2A). In elongated spermatids, nuclei were darker, polarized and nucleoli were absent (Figure 2A). In addition, 20.5% ± 1.2 of the cells collected after tissue digestion belonged to spermatid subpopulations, including round, early elongated and elongated spermatids (Figure 2B). Whereas, after completing the isolation procedure described in Figure 1, this value increased to 72.5% ± 2.2 (P < 0.05) (Figure 2B). In line with these results, FISH analysis showed that the percentage of haploid cells was on average equal to 76.7% ± 3.7 with respect to diploid cells (Figure 2C; Table 3).

**TABLE 3:** Assessment haploidy cells using fluorescent *in situ* hybridization. For each bull, percentage of spermatids, diploid cells and no signal are reported.

Sample	Spermatids (n) (%)	Diploid cells (2n) (%)	No signal (%)
Bull 1	74.0	11.0	15.0
Bull 2	72.0	21.0	7.0
Bull 3	67.0	21.0	12.0

#### 3.1.2 Evaluation of spermatid marker expression

**FIGURE 3:**
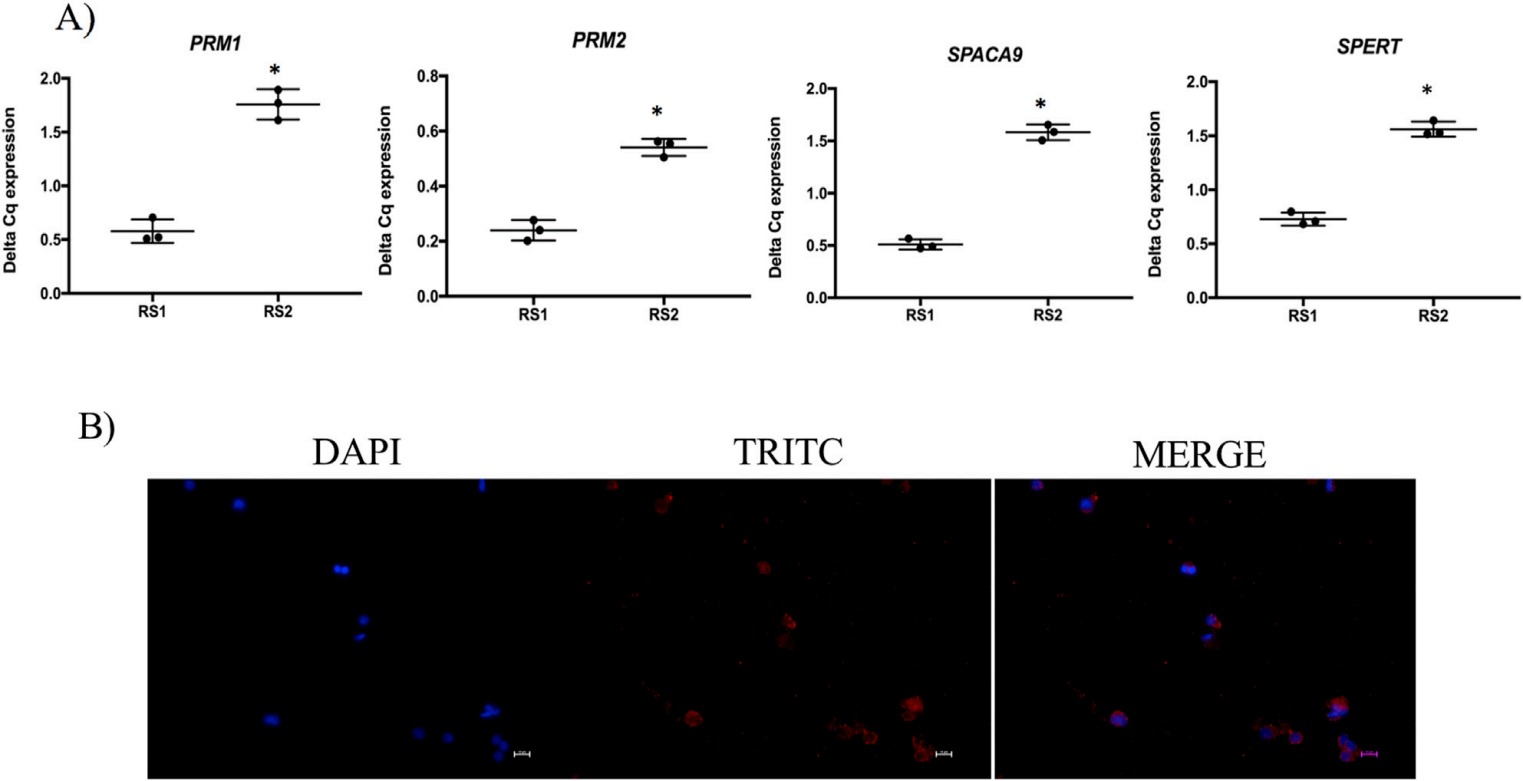
Gene and protein expression of spermatids. **(A)** Gene expression analysis of spermatid markers *PRM1*, *PRM2*, *SPACA9* and *SPERT* in the cells collected after enzymatic digestion of testicular parenchyma (RS1) and after carrying out the isolation procedure for purifying spermatids (RS2). **(B)** Representative immunofluorence microphotographs showing spermatid associated protein (SPERT) in the isolated spermatids, scale bar = 10 µm.

All spermatid cell markers analyzed, i.e., *PRM1*, *PRM2*, *SPERT* and *SPACA9*, were expressed in the cells obtained after tissue digestion (RS1) and in those collected after completing the isolating procedure (RS2). However, the expression level of all the transcripts was higher (*P* < 0.05) in the cells characterized by an enrichment in spermatid subpopulations (RS2), compared to the cells obtained right after tissue dissociation, confirming the efficiency of the procedure to increase the purity of collected spermatids (Figure 3A). In line with these results, SPERT protein expression was confirmed by immunofluorescence showing a specific cytoplasmic signal positivity in the spermatids (Figure 3B).

### 3.2 Culture and quality assessment of isolated spermatids

#### 3.2.1 Evaluation of viability and DNA fragmentation

**FIGURE 4:**
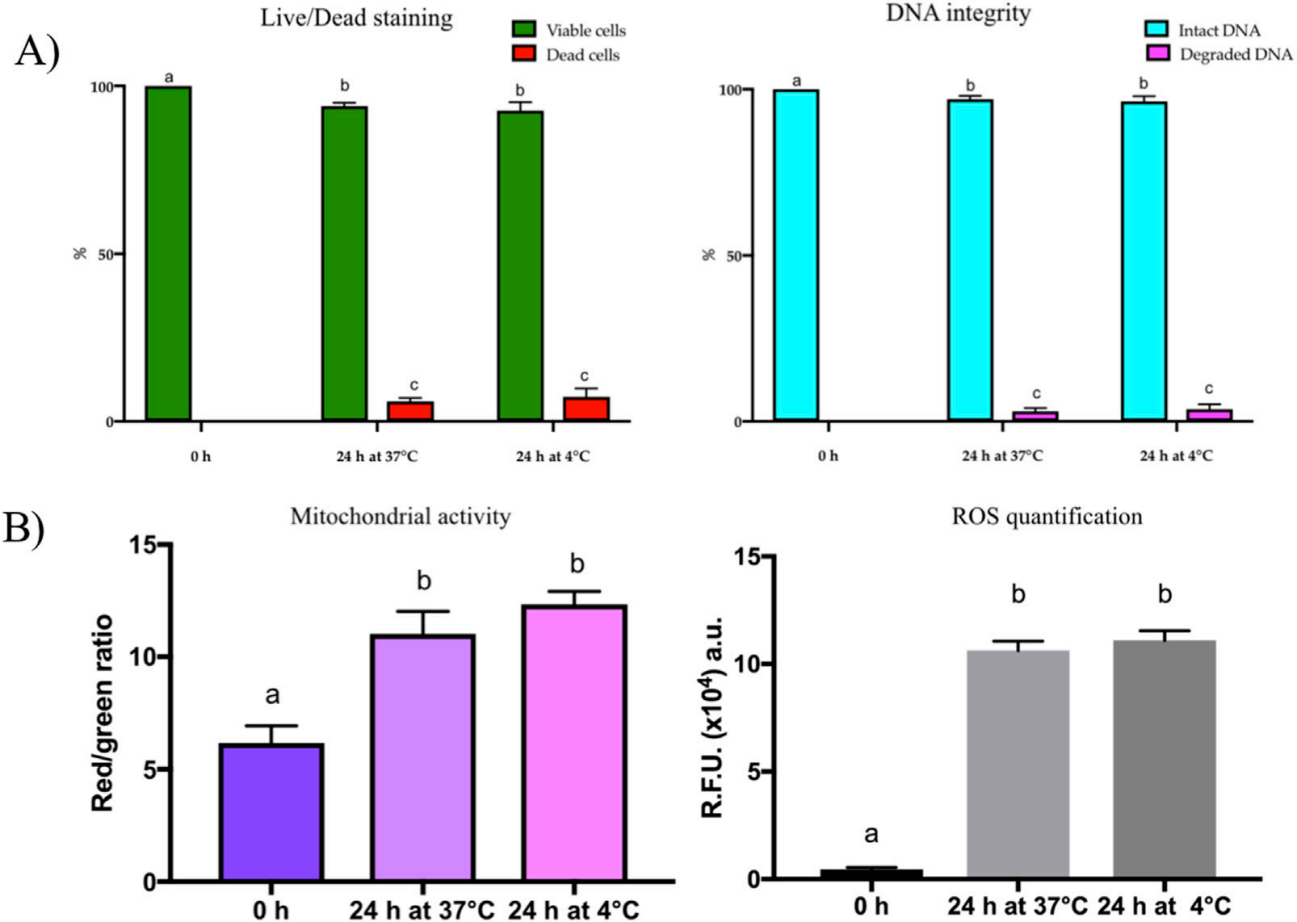
**(A)** Results of the viability (right) and DNA integrity (left) assays performed using LIVE/DEAD and acridine orange staining, respectively. Both assays were carried out immediately after round spermatid isolation (0 h) and after 24 h of culture at 37°C in 5% CO2 incubators (24 h at 37°C) and 4°C in air atmosphere (24 h at 4°C). **(B)** Results of mitochondrial activity (right) calculated as Red/green ratio and ROS quantification (left) immediately after round spermatid isolation (0 h) and after 24 h of culture at 37°C in 5% CO2 incubators (24 h at 37°C) and 4°C in air atmosphere (24 h at 4°C).

At 0 h, all cells were alive and had intact DNA (Figures 4A,B). At 24 h after culture, the percentage of alive cells was 98.2% ± 1.8% at 37°C and 97.8% ± 2.8% at 4°C (*P* > 0.05), respectively (Figure 4A, left). In agreement with these results, the percentage of cells with intact DNA was 99.0% ± 1.0% at 37°C and 98.5% ± 1.5% at 4°C (*P* > 0.05) (Figure 4A, right).

#### 3.2.2 Mitochondrial activity evaluation and ROS quantification

Mitochondrial activity significantly increased (*P* < 0.05) in the spermatids cultured for 24 h at 37°C and 4°C compared to those analyzed at 0 h (Figure 4B, left). We hypothesized that oxidative stress was occurring during *in vitro* culture of spermatids. In line with our hypothesis, ROS concentration was higher (*P* < 0.05) in the spermatids cultured for 24 h at 37°C and 4°C compared to those analyzed at 0 h (Figure 4B, right).

### 3.3 Spermatid fertilizing ability

**FIGURE 5:**
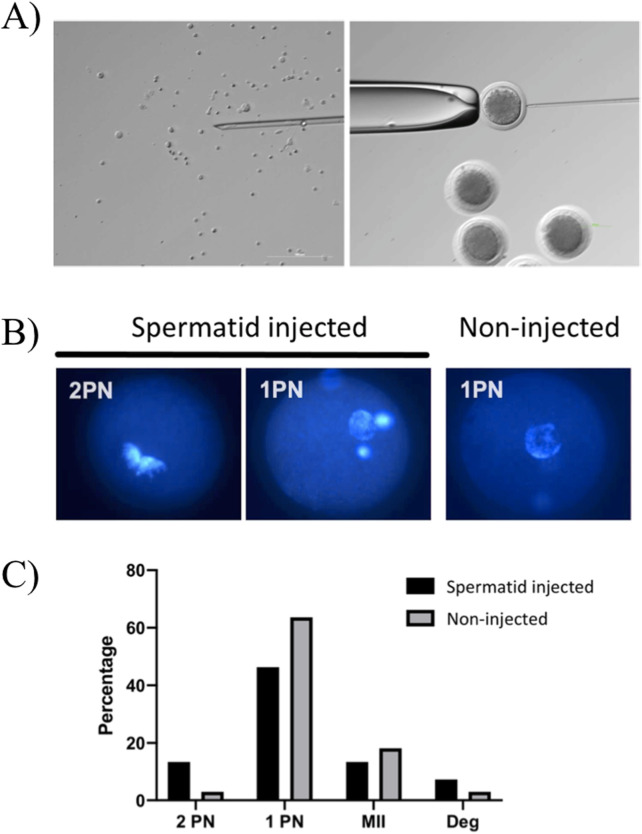
Spermatid injection in mature oocytes. **(A)** Representative images in bright field of spermatids ready to be injected (left) and injection in a mature oocyte (right). **(B)** Representative fluorescence images of zygotes with 2PN and 1PN in spermatid-injected and control, not injected oocytes. **(C)** Distribution of DNA configuration in spermatid-injected and control, not injected oocytes. Fishers’ chi-square exact test, no significant difference.

Figure 5A and supplemental video show the spermatid retrieval and oocyte injection. This procedure was carried out on 82 mature oocytes to assess the spermatid fertilizing ability. As graphically represented in Figures 5B,C, only 11 oocytes formed 2 PN upon activation (13.4%), while 38 displayed 1 PN (46.3%). Among the latter, 19 also showed a compact DNA spot within the cytoplasm that has been interpreted as the uncondensed spermatid. Notably, also non injected oocytes were sometimes able to form 2 PN, but at a much lower rate (1/33, approx. 3.0%), indicating that the majority of the 2 PN observed in the spermatid-injected group were bi-parental zygotes. The remaining oocytes were found either at the MII stage (n = 11), or at different/not interpretable stages (n = 16) or degenerated (n = 6).

### 3.4 Stage-specific spermatid isolation using flow cytometry

Cells positive for DNA staining were selected in the Gate 1. Thereafter, spermatids belonging to the spermatogenesis stages 1–12 were selected based on a dot plot showing granularity (SSC-A) *v*versus size (FSC-A) from gate 1. Then, spermatids from steps 1-9 and steps 10–12 were separated from each other according to the variation of DNA staining intensity (Gates 5 and 6). Spermatids from steps 13–14 and 15–16 were then selected from positively stained cells on a dot plot showing size (FSC-A) versus DNA staining (Alexa Fluor 488-A) represented by Gates 3 and 4. All sorted subpopulations were defined again with SYTO 16 FITC-W versus SYTO 16 FITC-A dot blots to boost their purity.

**FIGURE 6:**
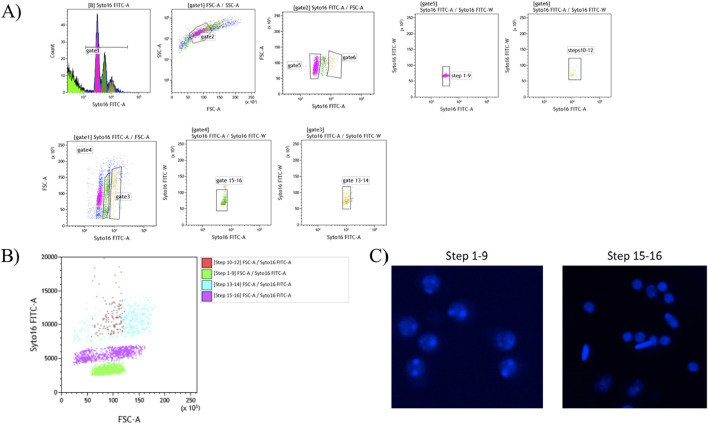
Flow cytometry approach for stage specific sorting of spermatid stage. **(A)** Gating strategy used with BD FacsMelody - Cell Sorter to sort pure spermatid subpopulations. **(B)** Flow cytometry data plots representing the steps used to isolate the spermatid subpopulations. **(C)** Representative microphotographs of steps 1–9 (right) and 15–16 (left) of the purified spermatid subpopulations.

Epifluorescence microscopy evaluation revealed that steps 1-9 were represented by a homogenous cell population of round spermatids, characterized by a round nucleus and the presence of nucleoli (Figures 6B,C, right). Whereas steps 15–16 were represented by an heterogenous cell population of elongating and elongated spermatids with small and elongated nuclei (Figures 6B,C, left). In contrast, in steps 10–12 and 13–14, spermatids were not efficiently separated (Figure 6B).

## 4 Discussion

In the present work, we developed a protocol for isolating bovine spermatids, achieving a significant enrichment of spermatid subpopulations in the collected cells. In line with these results, isolated cells showed high haploidy rate and transcript levels of spermatid markers (*PRM1*, *PRM2*, *SPACA9* and *SPERT*). Immunocytochemistry confirmed SPERT protein expression specifically in the cytoplasm of spermatid stages. Cells remained viable with intact DNA at 0 and 24 h of culture at 37°C and 4°C. However, cultured cells were characterized by increased mitochondrial activity and ROS levels. In addition, spermatids showed a low fertilizing ability after injection into *in vitro* matured oocytes. Notably, for the first time, we established a protocol for stage-specific isolation of bovine spermatids using a flow cytometry approach.

In the first phase of the isolation procedure, spermatids represented only 20.5% of the cells obtained from tissue digestion. At the end of the procedure, their percentage increased to 72.5%, indicating the method’s efficiency in selectively enriching for different spermatid stages. The methods for isolating spermatids have been mainly developed in mouse and include velocity sedimentation, density gradient (Bellve et al., 1977; Willison et al., 1990) centrifugal elution (Meistrich, 1977; Blanchard et al., 1991) immunoselection panning technique (Pelengaris and Moore, 1995) and BSA gradients (Kim et al., 2021; Li et al., 2024). In cattle, the use of Percoll density gradient resulted in only a total of 30%–40% of spermatids on the total cells isolated (Ock et al., 2006a; 2006b). Although all these procedures are not invasive for the cells, they are laborious and time-consuming. Here, we established a fast and easy-to-follow protocol, obtaining an efficient isolation of spermatids ready in only 1 hour that can be used for various other experiments. In addition, the morphological analysis confirmed the characteristics and composition of isolated spermatid subpopulations. Round and elongating/elongated spermatids were found to vary in size, ranging from 7.0 to 12.0 μm. In round spermatids, nuclei were centrally located with 1-3 nucleoli, while elongated spermatids exhibited darker, smaller or polarized nuclei lacking nucleoli. This distinction in nuclear and cell morphology aligns with the differentiation stages of spermatogenesis described in all mammalian species, including cattle (Staub and Johnson, 2018) and mice (Xu et al., 2021).

Supporting our results, FISH analysis further revealed that haploid cells were on average of a 76.7% of the total cell population isolated, once again confirming the efficiency of the procedure. These results also indicate the validity of the morphological identification approach that we performed to recognize all spermatid stages. In addition, these results were supported by the higher transcript level of all the spermatid markers analyzed, *PRM1* (Steger, 1999), *PRM2* (Hamilton et al., 2019), *SPERT* (Steger, 1999) and *SPACA9* (Chen et al., 2008), in the cells collected with our isolation protocol. Interestingly, SPERT protein immunopositivity was observed in our purified cells. We think this is an interesting aspect since SPERT was recently described to be distinctive of the elongation stages of the spermatids but absent in mature spermatozoa, in primates, pigs and rodents (Feige et al., 2002). Our results, detecting SPERT protein in the cytoplasm of isolated bovine spermatids, demonstrate that this protein is conserved among mammalian species and could, therefore, be used as a distinctive marker of these cell type in cattle as well.

The evaluation of spermatid quality using viability and DNA integrity assays revealed that, at 0 h, all cells were alive with intact plasma membrane and DNA. After 24 h of culture, viability remained high with few cells dead at both temperatures (37°C: 98.2% ± 1.8%; 4°C: 97.8% ± 2.8%). These values were closely mirrored by the proportion of cells with intact DNA (37°C: 99.0% ± 1.0%; 4°C: 98.5% ± 1.5%). Taken together, *in vitro* culture conditions used were able to preserve spermatids’ health as well as their genomic integrity. While we were expecting these results for cells cultured at 37°C, which is the standard temperature used for culturing mammalian cells, the results obtained from cells maintained at 4°C are intriguing. Our results are in agreement with (Martins et al., 2015) who described efficient spermatid preservation using refrigeration at 4°C. This suggests that spermatids can be preserved for 24 h, or even shipped to other laboratories, within this time frame before intracytoplasmic injection into mature oocytes.

It must be noted that culture spermatids showed significantly increased mitochondrial activity after 24 h compared to the 0-hour time point, regardless to the temperature. In the male reproductive system, mitochondria play a crucial role in developing and supporting germ cells, which are essential for producing healthy sperm (Costa et al., 2023). Several studies have shown that, *in vivo*, spermatogenic cells, including spermatids, require high consumption of lactate and glucose, metabolized through glycolytic and oxidative metabolism (Jutte et al., 1981; Bajpai et al., 1998). These molecules can enter the seminiferous tubules through Sertoli cells (Hall and Mita, 1984). Therefore, our findings may suggest that enhanced cellular metabolism during *in vitro* culture may result from tissue dissociation, which disrupts the interaction between Sertoli cells and spermatids. On the other hand, the described increase in metabolic activity is paralleled by an increase in ROS production in spermatids after 24 h, indicating the occurrence of oxidative stress. While mitochondrial activity is essential for cellular energy production, it is clear that excessive ROS levels can affect cell viability and function, potentially leading to oxidative damage over prolonged culture periods (Halliwell, 2003; Murphy et al., 2022). This suggests that further optimization of *in vitro* culture conditions may be beneficial to prevent oxidative stress and ensure long-term viability and function in the cultured spermatids. Future studies are mandatory to explore the use of antioxidants and/or other protective measures aimed at mitigating ROS accumulation while maintaining high viability and DNA integrity during extended culture time of spermatids.

Based on the results described above, we decided to perform intracytoplasmic spermatid injection into mature oocytes using spermatids immediately after their isolation.

The results of spermatid injection showed a rather low fertilizing ability, as only 13.4% of the mature oocytes formed 2 PN upon activation. Since the majority of the spermatid-injected oocytes went on to form 1 PN and few remained arrested at the MII, a possible interpretation is that the activation protocol is adequate to induce the maternal pronucleus formation. At the same time, spermatids fail to decondense in most of the cases. In line with this hypothesis, we often observed a spot of condensed DNA in the ooplasm of spermatid-injected oocytes along with 1 PN. We hypothesize that this DNA might derive from the injected spermatid, also because such a structure was absent in the non-injected, activated oocytes. Although confirmation using maternal and paternal pronuclei markers is still required, these initial results are encouraging, as they provide proof of concept that some bovine spermatids have acquired a certain degree of fertilization competence. Further studies characterizing subpopulations could help identify key differentiation events necessary to unlock this potential or reveal molecular signatures useful for selecting and culturing the most suitable spermatids.

Flow cytometry cell sorting results indicate that we successfully isolated two distinct subpopulations of bovine spermatids based on their apparent DNA content, size and granularity. Consistent with this, epifluorescence microscopy confirmed clear morphological distinctions of the nuclei in the isolated spermatid subpopulations, typical of later stages of spermiogenesis in mammals, including human (Tesarik and Mendoza, 1996), mouse (Hasegawa et al., 2010), and cattle (Staub and Johnson, 2018). Unfortunately, as described in the result section, steps 10–12 and 13–14 spermatid stages were not efficiently separated, indicating sorting inefficiency within these gates. One possible explanation for these results may be ethanol fixation of cells prior to sorting, which could somehow affect separation and isolation of elongating and elongated spermatids. Despite this, our results are in line with a recent study demonstrating efficient flow cytometry purification of round and elongating/elongated spermatids from human and rat testis, as well as a pure population of round spermatids from mouse testis using a DNA intercalating dye (Struijk et al., 2019). Although further optimization could enhance separation efficiency, the approach described here provides useful results for generation a robust tool to enable downstream analysis of gene expression, chromatin remodeling, and other stage-specific cellular processes in spermatid development and differentiation in cattle.

## 5 Conclusion

Overall, our results demonstrate that the procedure described in the present manuscript significantly enhances the isolation of viable and healthy bovine spermatids, defining morphological and molecular features for their proper identification and characterization. This approach is helpful for downstream applications in embryotechnology. In addition, although spermatid injection into mature oocytes remains unsuccessful in cattle, the flow cytometry protocol set up here is valuable for isolating highly purified spermatid subpopulations and for a better understanding of the functional and molecular aspects related to spermatid competence. Finally, this work provides useful data for developing spermatid injection protocols that might, in due course, allow to reduce the generational interval in cattle if competent spermatids are generated from embryonic cells.

## Data Availability

The original contributions presented in the study are included in the article/Supplementary Material, further inquiries can be directed to the corresponding author.
